# Using human-centered design to develop a nurse-to-family telehealth intervention for pediatric transfers

**DOI:** 10.1177/20552076231219123

**Published:** 2023-12-15

**Authors:** Jennifer L Rosenthal, Audriana Ketchersid, Elva Horath, April Sanders, Thomas A Harper, Adrienne E Hoyt-Austin, Sarah C Haynes

**Affiliations:** 1 Department of Pediatrics, 8789 University of California Davis, Sacramento, CA, USA; 2 Center for Health and Technology, 8789 University of California Davis, Sacramento, CA, USA

**Keywords:** Pediatrics, telemedicine, emergency medicine, nurses, patient transfer, human-centered design

## Abstract

**Objective:**

To develop a nurse-to-family telehealth intervention for pediatric inter-facility transfers using the human-centered design approach.

**Methods:**

We conducted the inspiration and ideation phases of a human-centered design process from July 2022 to December 2022. For the inspiration phase, we conducted a qualitative cross-sectional case study design over 3 months. We used thematic analysis with the framework approach of parent and provider interviews. Five team members individually coded transcripts and then met to discuss memos, update a construct summary sheet, and identify emerging themes. The team adapted themes into “How Might We” statements. For the ideation phase, multidisciplinary stakeholders brainstormed solutions to the “How Might We” statements in a design workshop. Workshop findings informed the design of a nurse-to-family telehealth intervention, which was iteratively revised over 2 months based on stakeholder feedback sessions.

**Results:**

We conducted interviews with nine parents, 11 nurses, and 13 physicians. Four themes emerged supporting the promise of a nurse-to-family telehealth intervention, the need to effectively communicate the intervention purpose, the value of a user-friendly workflow, and the essentiality of ensuring that diverse populations equitably benefit from the intervention. “How Might We” statements were discussed among 22 total workshop participants. Iterative adaptations were made to the intervention until feedback from workshop participants and 67 other stakeholders supported no further improvements were needed.

**Conclusion:**

Human-centered design phases facilitated stakeholder engagement in developing a nurse-to-family telehealth intervention. This intervention will be tested in an implementation phase as a feasibility and pilot trial.

## Introduction

Most emergency departments (EDs) lack pediatric admitting privileges or the necessary pediatric resources to provide definitive care to children who present with acute illness or injury.^1,2^ As a result, approximately 350,000 children in the United States are transferred between facilities each year, and the number of pediatric inter-facility transfers is rising over time.^3,4^ The transition of care from a referring ED to an accepting hospital involves both a change in the physical location of the child and a change in the care team members. This care transition, combined with the acute condition of the pediatric patient, can cause stress to both the child and their parent or guardian (“parent” hereafter).^5,6^ Pediatric inter-facility transfers can also involve communication breakdowns^6^ that can impact patient safety and hinder parent experience.^7–9^ Lack of shared decision-making between parents and providers worsens parents’ stress and exacerbates communication disconnects.^10^

To mitigate potential harm to patients and parents during inter-facility transfers, effective communication, and engagement among providers and parents is critical. The use of telehealth during inter-facility transfers to bring an accepting provider virtually to the child's bedside in the referring ED is a promising strategy to enhance parent–provider communication and parent engagement.^11^ Prior research conducted at our children's hospital has demonstrated benefits of telehealth use by our hospital's post-transfer accepting physicians and nurses during pediatric ED encounters.^12–15^ These prior studies examined provider–provider telehealth communication, whereby the parent can be (optionally) included in the virtual connection. Research is lacking that examines an alternative model that is more parent-centric. In this alternative model, the accepting provider connects virtually with the parent in the referring ED, and the referring providers can be (optionally) included in the virtual connection.

To address this research gap, our team set out to develop a provider–parent telehealth intervention by actively involving stakeholders in the design process. Human-centered design (HCD) is a stakeholder engagement approach that can be used in healthcare research.^16–18^ When using HCD, researchers include individuals from the population of focus to help create solutions to address specific problems or needs.^19^ The HCD process includes three phases: inspiration, ideation, and implementation.^19^ The inspiration phase includes the creation of ideas and solutions by engaging a variety of people. The ideation phase includes sharing all discoveries with a team, analyzing the data, and coming up with opportunities to enhance the project. Finally, in the implementation phase, the solutions that were created by the team are tested. This process can involve iterative cycles of testing and improving to optimize the user-centric solutions.

This paper discusses the HCD approach used to inform the protocol for a feasibility and pilot trial comparing nurse-to-family telehealth communication to standard of care for pediatric patients being transferred between hospitals. The purpose of this article is to describe the procedures and findings of the inspiration and ideation phases of HCD.

## Methods

### Overview

**Figure 1.**
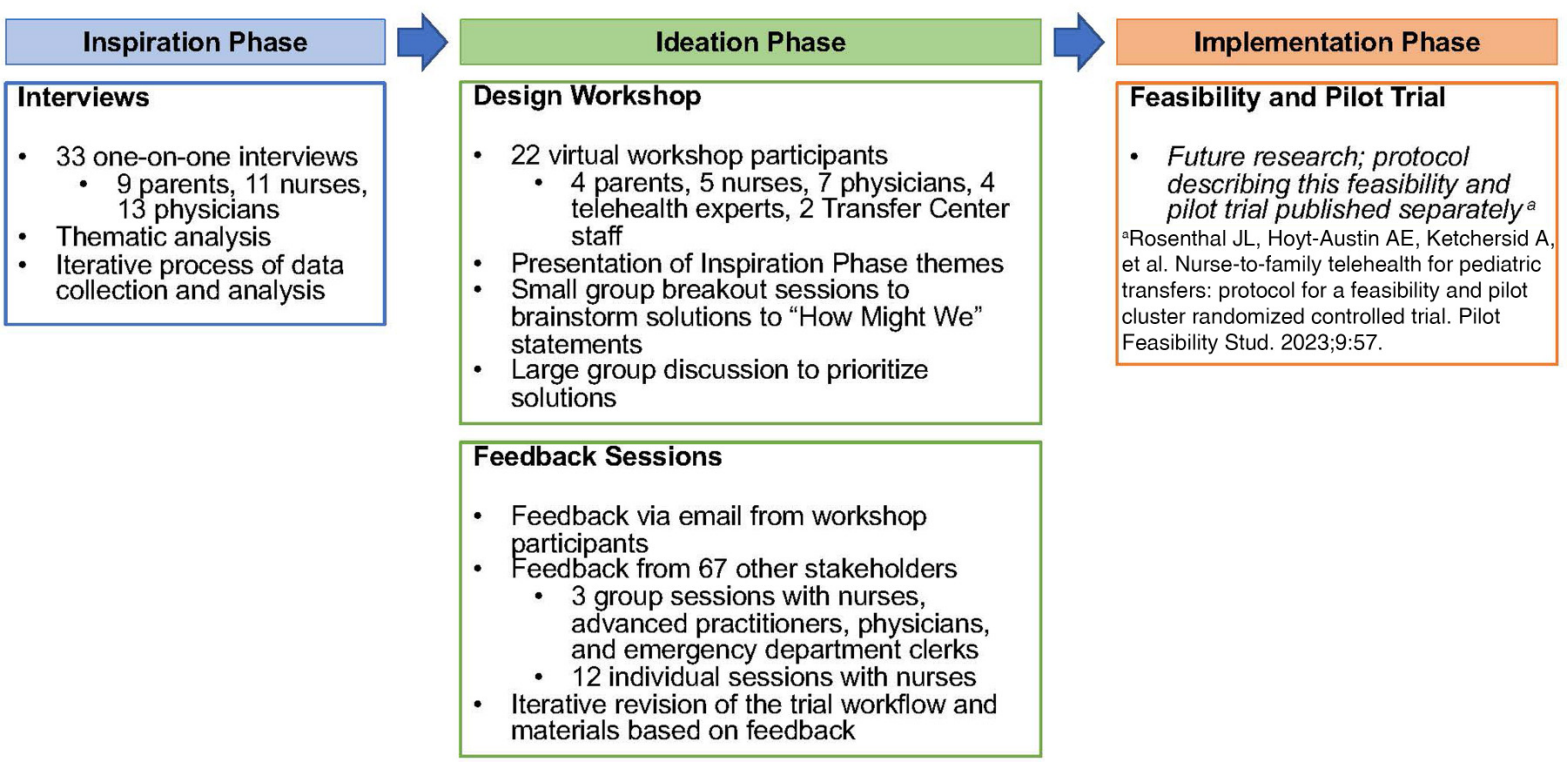
Overview of the activities comprising the human-centered design phases.

A core team was convened to lead the inspiration and ideation phase HCD procedures from July 2022 to December 2022. This team was comprised of two research analysts, a pediatric hospitalist, a telehealth researcher, a general pediatrician, and a telehealth program manager. The inspiration phase consisted of a qualitative study using a cross-sectional case study design.^20^ The cases of interests were events, specifically parents and providers who experience the event of caring for a child during an inter-facility transfer. Over a period of 3 months, we used semi-structured interviews and thematic analysis with the framework approach.^21^ The ideation phase consisted of a stakeholder design workshop followed by stakeholder feedback meetings with iterative improvements over a 2-month period. The qualitative findings from the inspiration phase informed the content of the workshop in the ideation phase. An overview of the HCD phases is shown in Figure 1.

Eligibility requirements for participation in interviews, the workshop, and feedback sessions included being at least 18 years old and having English language proficiency. Participants had to be a parent or guardian, nurse, physician, or telehealth expert. Parents and guardians had to have experienced an inter-facility transfer of their child. Nurses, physicians, and telehealth experts had to work at either the post-transfer hospital or a referring ED site; they had to have a current role in caring for transferring children or facilitating the use of telehealth for pediatric transfers. We excluded individuals’ age less than 18 years, because the focus of the study was to design an intervention that would be used by parents and providers. Thus, the perspectives of children in designing this intervention were beyond the scope of this present study. The number of individuals who participated in each human-centered design phase was determined differently for each phase. For the inspiration phase, sample size of interviewees was based on reaching thematic saturation. For the ideation phase workshop, we aimed to have 20–25 stakeholders to allow for a diversity of perspectives while still maintaining a group size conducive to active participation. For ideation phase feedback sessions, we continued soliciting feedback from stakeholders until no further improvements were identified.

The inspiration phase of this study was approved by the University of California Davis Institutional Review Board. The ideation phase – which did not meet the definition of research involving human subjects – was exempt from Institutional Review Board review.

### Setting

The post-transfer, accepting hospital is a metropolitan tertiary care children's hospital within a university hospital. This 129-bed children's hospital is in Northern California. It is the referral center for children across a 33-county region covering 65,000 square miles. The children's hospital receives transfers from over 130 EDs and hospitals in the region and accepts over 2500 patients as transfers annually.

### Inspiration phase: interviews

#### Data collection

The inspiration phase used one-on-one interviews with parents, physicians, and nurses. We used convenience sampling and snowball sampling to recruit participants. Eligible parents were those with a child who experienced an ED-to-inpatient inter-facility transfer. A core team member recruited parent participants in-person during the post-transfer hospitalization and obtained informed consent. Parent interviews were conducted in-person; the hospitalized child was present for most parent interviews. Eligible physicians and nurses were pre-transfer ED providers or post-transfer children's hospital providers. Physicians and nurses were recruited by email or in-person. Physician and nurse interviews were conducted either in-person or via videoconference. Sampling continued until we reached thematic saturation.^21^ We conducted 33 total interviews, including nine parents, 11 nurses, and 13 physicians. Most interviewees identified as not Hispanic/Latino, identified as female, and were 31–40 years of age. Interviewee characteristics are shown in Table 1.

**Table 1.** Interview participant characteristics.

	Role		
Characteristic	Parents (*n* = 9)	Nurse (*n* = 11)	Physicians (*n* = 13)
Race, *n* (%):			
Multiracial	0 (0)	2 (18.2)	2 (15.4)
White	4 (44.4)	8 (72.8)	5 (38.5)
Asian	2 (22.2)	1 (9.1)	5 (38.5)
Other	3 (33.3)	0 (0)	1 (7.7)
Ethnicity, *n* (%):			
Hispanic	3 (33.3)	3 (27.3)	0 (0)
Not Hispanic	6 (66.7)	8 (72.7)	13 (100)
Age (years), *n* (%):			
20–30	1 (11.1)	2 (18.2)	2 (15.4)
31–40	6 (66.7)	6 (54.5)	8 (61.5)
41–50	0 (0)	1 (9.1)	2 (15.4)
51–60	2 (22.2)	2 (18.2)	1 (7.7)
Gender, *n* (%)			
Male	2 (22.2)	0 (0)	3 (23.1)
Female	7 (77.8)	11 (100)	10 (76.9)
Highest education, *n* (%)			
Less than high school graduate	2 (22.2)		
High school graduate	2 (22.2)		
Some college	2 (22.2)		
2-year college degree	2 (22.2)		
4-year college degree	1 (11.1)		
Years of experience after training (years), *n* (%)			
0–5		3 (27.3)	2 (15.4)
6–10		0 (0)	6 (46.2)
11–15		2 (18.2)	0 (0)
16–20		3 (27.3)	0 (0)
21+		2 (18.2)	2 (15.4)
Missing		1 (9.1)	3 (23.1)
Comfort level using telehealth, *n* (%)			
Very uncomfortable	0 (0)	1 (9.1)	0 (0)
Slightly uncomfortable	0 (0)	3 (27.3)	3 (23.1)
Neutral	2 (22.2)	2 (18.2)	5 (38.5)
Slightly comfortable	1 (11.1)	3 (27.3)	2 (15.4)
Very comfortable	6 (66.7)	2 (18.2)	3 (23.1)

A core team member (AK) conducted the interviews using a semi-structured interview guide to explore domains described in the Consolidated Framework for Implementation Research (CFIR).^22^ The CFIR is a widely used determinant framework to assess contextual factors that can influence the outcome of implementation efforts. The interview guide solicited information on the following CFIR constructs: innovation (evidence base, relative advantage, complexity), financing, pressure (external, performance measurement), infrastructure (physical, information technology), compatibility, available resources, access to knowledge and information, need, motivation, and assessing context. The interview guide was not tested prior to use. Interviews were audio recorded, professionally transcribed, and de-identified. Interview participants received a $50 gift card.

#### Analysis

Data collection and analysis occurred concurrently. Data were analyzed using thematic analysis following the principles of Braun and Clark's approach^21^ with the framework approach.^23^ The framework utilized the CFIR domains. Five team members independently performed open coding and analytic memo writing of the transcripts to identify potential findings that did not fit into the predetermined framework categories. These researchers agreed that the framework adequately represented the major findings. Subsequently, the team independently memoed and manually coded; the deductive codes pertained to the CFIR constructs. The team then met as a team to ensure consensus on application of codes, develop tentative categories, and examine the data for patterns and variations. A participants’ role-ordered matrix was created to explore relationships between constructs and participants’ role types. Tentative hypotheses were formulated regarding relationships among categories. The team searched for negative and qualifying evidence and adapted the interview guide to further explore topics. Recurrent unifying concepts and identified linkages and patterns between the categories became analytic themes. We worded the themes as insight statements. Insight statements are used in human-centered design to represent user perspectives, motivations, and tensions.^19^ We used ATLAS.ti to organize and store coding and data analysis.^24^

### Ideation phase: stakeholder design workshop

The ideation phase began with a stakeholder design workshop. Workshop participants were identified by suggestions from interview participants and recruited by email. Participants included parents, nurses, physicians, telehealth experts, and hospital transfer center staff. We purposively recruited participants representing diversity in roles and hospitals. The workshop was held via videoconference to promote participation from diverse participants, including those residing in remote locations or those with competing work or home responsibilities. Participants received a $100 gift card for this 2-hour workshop. The compensation of $50 per hour was offered to stakeholders to acknowledge the time they dedicated, recognize their valuable expertise, and offset any potential expenses incurred from their participation.

A core team member (JLR) with experience facilitating stakeholder engagement sessions led the workshop; other core team members facilitated the workshop small group sessions. The workshop began with core team and participant introductions, followed by explanation of the value of stakeholder engagement and human-centered design. We discussed principles of effective communication to promote learning about each other's priorities and ensuring all partners’ voices were heard and respected. We shared that the goal of the workshop was to work together to develop strategies that will be used for a future pediatric inter-facility transfer trial; the future trial purpose was to improve care delivery by making pediatric hospital-to-hospital transfers more family-centered.

We subsequently presented an overview of the knowledge gained from the inspiration phase. Specifically, we shared the insight statements (i.e., qualitative themes) and corresponding “How Might We” (HMW) statements that were developed by the core team. Adapting insight statements into HMW statements is a strategy used in human-centered design to create brainstorming prompts phrased as questions.^19^ In doing so, workshop participants could generate solutions to the insights that were generated during the prior inspiration phase.

We divided the workshop participants into three small groups of seven or eight stakeholders for the brainstorming session. Individuals used Google Jamboard to brainstorm their solutions to the HMW statements. The small groups discussed the generated ideas and then revised and added to the ideas. Workshop participants rejoined as a larger group to report back, discuss solutions, and categorize and prioritize the solutions. To prioritize the solutions, each stakeholder was asked to select their top 3 solutions they perceived should be prioritized in designing the feasibility and pilot trial. Specifically, stakeholders anonymously placed images of award ribbons over their top3 solutions. The number of ribbons per strategy was counted to determine perceived priority.

### Ideation phase: stakeholder feedback sessions

After the design workshop, the core team incorporated the stakeholders’ solutions to draft trial workflow and materials to be tested in a future implementation phase. Feedback was solicited from workshop participants and subsequently from additional nurse and physician providers. Iterative changes were made, and stakeholders were invited to provide feedback, until feedback supported that no further adaptations were needed to improve the workflow and materials.

## Results

### Inspiration phase: interviews

We identified four themes from the inspiration phase interviews. Each theme, worded as an insight statement, is presented in Table 2, along with categories and supporting quotes. These themes are also further explored below.

**Table 2.** Themes and supporting quotes.

Theme	Categories	Supporting quotes
**Theme 1:** Virtually connecting receiving providers to parents before transfer is a promising intervention	Telehealth can prepare parents for the transfer process	“Just being able to know the next steps and a timeframe of what happens, actually… I had to follow them in an ambulance. It would have been nice to know where am I supposed to park? What things do I bring with me? What things are okay to go back to the car and get? Almost logistical stuff more than the medical questions.” – Parent (23)
		“…our policies, like visitor policies and stuff like that, haven’t been explained. So, the parent may come with extra family members that aren’t allowed on our floor at the moment or things like that, and that can cause a lot of frustration for the families as well… I think [telehealth] could help a lot because we could explain… explaining all of that before they come I think is helpful because they can kind of prepare, especially if they’re coming from far away.” – Nurse (20)
	Telehealth can provide support to parents during a stressful event	“In this case we were at a hospital that did not really specialize in dealing with children… We were limited conversing just with the personnel they had there. And they would admit that, ‘We’re kind of limited here, so we want to get him transferred.’ So I think [telehealth] would give you more reassurance… you could get some confirmation of that and you would feel more comfortable.” – Parent (08)
		“I think [telehealth] sounds promising, and there have been many times like that the transfer will come in, and they have no idea what to expect when they arrive to the floor, and that can cause a lot of anxiety for families… [Telehealth] just creates more like introduction to the unit, and to patients who may have not been [here] before. So I think this could be beneficial.” – Nurse (30)
	Telehealth can prepare receiving nurses for the patient's arrival	“… how they feel that their child is doing would also be helpful, because a parent's perspective is always super important. Looking through the intake on transfers isn’t always super clear as to what their child may or may not look like, so getting [the parent's] input also might be helpful.” – Nurse (30)
		“I think that it will be helpful, especially from a nursing perspective because we generally are the first part of the team that greets these patients coming to the floor. So, being able to have that initial meeting virtually I think can help make that transition a little bit easier… questions about the patient specifically if there are other diagnoses or certain things or the patient might need certain accommodations.” – Nurse (33)
**Theme 2:** Clearly messaging the purpose and expectations of the telehealth visits is necessary to avoid unintended consequences	Distinguishing that these telehealth visits are a new service that is separate from existing physician telehealth consultations	“When you say ‘telehealth,’ people are thinking, ‘I’m going to get a medical consultation’… I could see a mismatch between what their expectation is and what we’re set up to do. And so it might be helpful to sort of publicize what we’re there for.” – Pediatric Attending (17)
		“My initial thoughts are mostly questions about what the purpose of this would be. Would it be in addition to [the existing program] – so would it be something that we would talk with the transferring facility physician or a health provider and then communicate directly with the family?” – Pediatric Attending (14)
	Communicating a meaningful, relevant purpose of the telehealth visits	"People will do something if they feel like it is helping something… there's a potential that [telehealth] could improve for a few minutes or a few hours parental quality of life, and does that equate to some health outcome or patient-centeredness outcome that matters clinically?” – Pediatric Attending (09)
		“[Telehealth] allows them to be able to participate in situations… and that makes me as a provider feel more comfortable going forward with care plans and such, in order to make sure that we have the family's input to make sure they're on board.” – Nurse (12)
	Setting clear expectations for the telehealth visit	“I think it just kind of depends on what the expectation is, and whether or not it's clearly communicated to families. Like, ‘You can expect to be contacted at this time, and then a reasonable responsible would be within this many minutes’… I think it kind of depends, too, on what the family is like. If they’re more understanding and willing to wait… or if they want the response right now.” – Nurse (04)
		“If we are so busy on the unit, or something's happening on the unit, and we don't have time to contact those parents before transport's there, then we’re going to miss that opportunity… the timing would just have to line up right. So that's the only thing that I worry about is timing… we definitely would make it a priority, but there's going to be the timing…” – Nurse (32)
**Theme 3:** Creating a user-friendly workflow is necessary to minimize provider burden in order to increase adoption	Competing demands and challenges to taking on additional tasks	“I definitely see the benefit behind it. At the same time, I guess the first things that come up is just time… The bedside nurses are extremely stressed right now… Adding one more thing to their plate might be a little bit too much.” – Nurse (28)
		“I would say more time or dedicated – somebody who's dedicated to doing that… to do all of this telehealth… from a resource perspective, that's what I would advocate for.” – Pediatric Attending (14)
	Streamlined workflows to avoid delays	“Procedurally, how will we get the parent's cell phone number? Will that be something that we then have to ask the [hospital] physician to go get the parent's cell phone number?… Who is logistically going to collect that information and get it to us? I could see that causing some delays or some frustration on the part of the other provider.” – Pediatric Attending (17)
		“I think one of the biggest barriers at this point is really that we have multiple or at least two different ways of making that [telehealth] connection… I’m never sure if I’m going to be able to connect or how efficiently I’m going to be able to connect… anything that can streamline those workflows is great.” – Pediatric Attending (18)
	Needed resources to support the use of telehealth	“You just need clear protocol as to when to initiate it… group here likes very clear protocols on when to initiate and not, and we have a unit clerk who does that, too. So, as long as they know when to do it, that's all they need.” – ED Physician (25)
		“We don’t have a private space… there is no private room, or office that can be used even briefly used for this type of visit.” – Nurse (29)
	Charge nurses are best suited for the role of conducting a provider-to-parent telehealth visit	“Right now usually our charge nurses are the nurses that are in charge of reviewing the charts prior to our patients coming up to our floor. Usually, they gather all the information. I know sometimes when we have direct transfers from home to our unit, our charge nurses are the ones that call the parent and say, ‘Hey, the bed's ready,’ and can give them extra information. It might actually be beneficial for the charge nurse to give the [telehealth] call.” – Nurse (20)
		“I do think that it would be worthwhile to maybe have the charge nurses specifically be the nurses that would be connecting with families. I think that bedside nurses just kind of are generally always dealing with some type of time constraint… it might be helpful to have the charge nurse who generally has a little bit more wiggle room in their schedule to be the first point of contact.” – Nurse (33)
**Theme 4:** Ensuring the intervention equitably benefits diverse populations is a priority to all users	Barriers for those who speak a language other than English	“Half my floor speaks Spanish right now, so I’d be interested to see how you would incorporate that… I don’t know if there would be an interpreter on the screen with them or how that would go.” – Nurse
		“Will we have the ability to on the spot get interpretation in all languages that we would need, or is this something that's going to be made available only to patients who speak English?” – Pediatric Attending (09)
	Lack of a functioning smart phone	“As long as you have a phone. Not everybody has a cell phone.” – Parent (23)
		“Technology barriers, so whether or not they have the appropriate phone, or they’re technology-savvy and know how to do it.” – Nurse (04)
	Poor or no cellular/Internet connection	“…if the person doesn’t have a phone, what do you do, or if there's not a good Wi-Fi connection, or they cannot, for some reason connects to the Wi-Fi, and in our ER we don’t have any cell phone coverage, so it’d have to be through Wi-Fi, and occasionally that goes down.” – ED Physician (26)
		“I suppose you’re relying on them having the infrastructure like a cell phone. Most people have cell phones, but a data plan that’ll support that. Is there Wi-Fi at that place? How fast is it?… I don’t know how many technical difficulties there may be in terms of the bandwidth of the Internet.” – Pediatric Attending (19)
	Low digital literacy, health literacy, and literacy	“…even them reading, if they read, or if they write. That can be hard, too.” – Nurse (04)
		“… if there's other barriers like not having access to a smartphone device or if they’re just not very comfortable using electronic devices. If they’re just not comfortable using electronic devices like that, if they’re not very tech-savvy, I could see those issues happening.” – Nurse (20)

ED: emergency department.

*Theme 1: Virtually connecting receiving providers to parents before transfer is a promising intervention.* Parents and providers universally perceived that telehealth use during pediatric inter-facility transfers could help prepare parents for the transfer process. Parents explained how virtual connections would be very helpful for them to be aware of the next steps and to understand the logistics surrounding the transfer process and procedures. Example topics shared by parents included what items to bring to the hospital, where to park, and COVID-19 vaccination policies. Nurse participants similarly mentioned that telehealth would help prepare parents. The most expressed benefit shared by nurses was about informing parents of the visitation policy that prohibited children visitors.

Parents, nurses, and physicians also shared how telehealth could provide support to parents during a stressful event. Participants spoke about ways in which telehealth use during a transfer could strengthen parent–provider relationships. Nurses explained how connecting with parents before the patient and family arrives at the post-transfer hospital would also be beneficial for the nurses, as they could better prepare for the child's arrival by gathering additional history and information that is not shared during the nurse-to-nurse handoff. Nurses explained how a nurse-to-family telehealth visit would be especially helpful for situations when parents are unable to be physically present with the child during transport; nurses could learn from parents how to best support the child's unique needs.

*Theme 2: Clearly messaging the purpose and expectations of the telehealth visits is necessary to avoid unintended consequences.* Many physician participants expressed the need to distinguish this nurse-to-family telehealth intervention from existing telehealth services. Multiple physicians raised concern that a new workflow to implement a nurse-to-family telehealth intervention could trigger confusion given the existing physician-to-physician telehealth consultations that could be requested for pediatric inter-facility transfers. Participants emphasized the importance of clearly communicating that the nurse-to-family telehealth intervention was an additional resource to the existing physician-to-physician telehealth consultations. Nurses and physicians raised concern that parents and providers might misunderstand that these nurse-to-family telehealth visits were replacing physician-to-physician telehealth consultations. They expressed concern that parents might expect nurses to provide medical advice during the telehealth connection; however, expectations should be established that medical decision-making is beyond the scope of this intervention.

Providers also highlighted the importance of anticipating that some pediatric inter-facility transfers might not be conducive to a nurse-to-family telehealth visit. The timing of the child leaving the pre-transfer hospital and the availability of the post-transfer provider were two commonly mentioned issues that could impede a nurse-to-family telehealth visit. Participants explained that it would be crucial to anticipate such potential problems and create workflows that account for dynamic and unique circumstances to minimize unintended negative effects.

*Theme 3: Creating a user-friendly workflow is necessary to minimize provider burden in order to increase adoption.* Nurse and physician providers unanimously stressed the importance of implementing the nurse-to-family telehealth intervention without imposing additional burdens on the pediatric providers. Providers shared how competing demands have left the nurses with limited ability to take on additional tasks. Therefore, adoption of the telehealth intervention would remain low unless workflows could be streamlined. Additional resources that providers mentioned as requirements to support a nurse-to-family telehealth intervention were dedicated equipment such as video-enabled computers and headsets. Providers also highlighted the need for quiet space to conduct the visits. While some providers identified the need for private locations to minimize interruptions and distractions, other providers identified the need to conduct the telehealth visits in a location that was not isolated but rather allowed them to remain accessible and visible to team members.

Regarding the ideal post-transfer provider to conduct the telehealth visit with the parents, multiple nurses and physicians suggested the receiving unit's charge nurse. Nurse participants explained that, in current practice, charge nurses review the available information about the transferring child prior to their arrival. Charge nurses also speak by telephone with parents of children being admitted from home. They are also most knowledgeable about their hospital unit's policies and procedures and thus best positioned to share such relevant information with the parents of transferring children.

*Theme 4: Ensuring the intervention equitably benefits diverse populations is a priority to all users.* Parents and providers ubiquitously expressed various concerns about the equitable reach and impact of a nurse-to-family telehealth intervention for families with a language preference other than English. Participants stated the importance of including medical interpreters for telehealth connections, when applicable. Some participants identified the potential challenges of parents navigating the English language telehealth platform. Multiple participants also expressed concerns about digital literacy, health literacy, and literacy impacting parents’ ability to access this intervention. Lack of a functioning smart phone was another stated barrier. Furthermore, poor cell phone service and no Internet connection were additional identified challenges. Providers spoke about locations in their ED that lacked cellular service. Other providers spoke about having free Internet in their ED, but how parents might need assistance in connecting to the Internet. A universal perspective shared by all participants was the need to address these access issues to optimize equitable reach and impact across our diverse groups of patients and families.

### Ideation phase: stakeholder design workshop

Using the insight statements from the inspiration phase, the core team developed three HMW statements. These statements asked the following: (1) HMW best explains these nurse–parent telehealth visits to everyone, (2) HMW creates a user-friendly workflow, and (3) HMW delivers telehealth equitably. We presented these statements to the 22 workshop participants. Participants included four parents, five nurses, seven physicians, four telehealth experts, and two transfer center staff representing four different hospitals. The HMW statements and corresponding solutions developed by the stakeholders are shown in Table 3.

**Table 3.** Workshop How Might We statements with corresponding proposed solutions.

How Might We statement	Categories	Proposed solutions
How Might We best explain these nurse–parent TH visits to everyone?	What is the purpose of the TH visits?	To provide clarity about transfer process To answer any questions To establish a relationship with the receiving charge nurse
	How should the TH visits be introduced to parents?	Family-facing information sheet about the TH visit QR code for family to scan to learn more about the TH visit List of topics that can be discussed during the TH visit Script for the emergency department providers to explain the purpose of TH visit to families
	How should providers be taught/trained?	Provider-facing information sheet about the TH visit QR code for providers to scan to learn more about the TH visit and for just-in-time training Create provider outline/guide of steps to conducting a TH visit Advertise the 24/7 TH help desk to providers Offer multiple training options (video, tip sheet, group instruction, one-on-one instruction) Resource person available if a refresher is needed
	Avoid confusion with existing physician-physician TH visits	Refer to TH visits as “nurse-to-parent” visits Avoid the term “telehealth;” replace it with “video chat” to suggest this TH intervention is different from existing physician–physician TH visits used for clinical decision-making
How might we create a user-friendly workflow?	Some parents may not want a TH visit	Include on family-facing information sheet a mechanism for family to opt in or out of the TH visit Explain the potential benefits of the TH visit to the parent
	The charge nurse may be unavailable	Send parent text message offering TH visit and ask them to reply “yes” if interested; if so, charge nurse responds with time frame Designate a back-up nurse if charge nurse is unavailable Provide families a timeline for the TH visit process Explicitly state to family that a charge nurse might not always be available to conduct a TH visit
	The charge nurse needs the parents’ contact information	Include on the family-facing information sheet a place to write family's contact information Collect family contact information for TH visit at ED check-in Transfer center collect family contact information for TH visit
	A quiet location and equipment is needed for the TH visit	Headphones with microphone for charge nurse Designate a private space for charge nurse Charge nurse initiates TH visit from a computer on wheels in the room the patient is assigned
	Timing of the TH visit	Charge nurse responds with timeframe for TH visit initiation Give families specific timeline for when TH visit will occur Transfer center ask charge nurse if they are available prior to offering TH visit to family Initiate TH visit as soon as a bed has been assigned Keep TH visit brief (∼5 minutes)
How Might We deliver TH equitably?	Lack cell phone or poor service	Provide families with tablets Utilize a landline in the ED as an option if TH visit is not possible
	Different levels of digital skills	Family-facing sheet with images and step-by-step instructions on how to join the TH visit Invite family to join TH visit via both email and text message Provide families with tablets already connected to ED Wi-Fi

TH: telehealth; ED: emergency department.

### Ideation phase: stakeholder feedback sessions

The core team used the solutions from the workshop to draft a flow diagram of the proposed telehealth intervention workflow as well as a parent-facing informational flyer. The flow diagram and parent flyer were distributed by email to the workshop participants. Feedback suggesting adaptations was received from a children's hospital charge nurse, telehealth expert, and transfer center staff. Concerns about the workflow were raised regarding the use of a fax machine as the way for pre-transfer ED staff to communicate to the children's hospital charge nurse that a parent wanted a telehealth visit. In response, we added to the workflow a phone call to notify the children's hospital charge nurse that a fax was being sent. Another suggestion that we incorporated was to specify that the charge nurse should wait at least 5 minutes after initiating the telehealth connection for the parent to join the visit. Regarding the flyer, we received and incorporated feedback on wording of the text and feedback to add a picture of a nurse using telehealth with a family. We emailed the revised versions of the flow diagram and parent flyer to the workshop participants. Feedback suggesting adaptations was received from a parent and a pre-transfer physician confirming that no further adaptations were needed.

In response to the workshop solution to offer multiple training options, the core team drafted two versions of training videos and written instructions, one for the pre-transfer ED providers and one for the post-transfer children's hospital providers. We subsequently solicited feedback and iteratively made improvements to the trial workflow, parent flyer, and training materials. Feedback was obtained from 67 total stakeholders. We conducted three group feedback sessions with pre-transfer ED nurses, advanced practitioners, physicians, and clerks. We also conducted 12 individual sessions with post-transfer children's hospital charge nurses. Feedback received and incorporated included providing children's hospital charge nurses with a list of potential topics to discuss with parents during the telehealth visits. In response to feedback, we also conducted additional trainings with pre-transfer ED unit clerks. No further improvements to the trial workflow and materials were deemed necessary. The resulting trial protocol was registered in ClinicalTrials.gov (NCT05593900) and published separately.^25^

## Discussion

Telehealth use during pediatric inter-facility transfers is a promising intervention to promote more family-centered care and to mitigate parent distress and triage issues. The optimal design and implementation of a telehealth intervention that brings a pediatric provider virtually to the child's bedside in the ED depend on local contextual factors. These factors include, but are not limited to, components such as infrastructure, available resources, providers’ motivation, and parents’ needs and values. In this study, we engaged stakeholders in the inspiration and ideation phases of a HCD approach. In doing so, we gained valuable information from individuals with diverse perspectives who represent the people – parents and providers – that the telehealth intervention is intended to benefit. Importantly, these stakeholders represent the providers who will be involved in implementing the intervention. Including a variety of stakeholders in the iterative design procedures unveiled potential barriers that were unforeseen by the core research team alone. Our HCD approach facilitated the design of a feasibility and pilot trial comparing nurse-to-family telehealth communication to standard of care for pediatric patients being transferred between hospitals.^25^ This feasibility and pilot trial is the next step for this research and represents the implementation phase of HCD.

Themes from the inspiration phase aligned with existing telehealth research. The insight that the intervention workflow must minimize provider burden is a topic that presents repeatedly in the telehealth literature. Previous qualitative and mixed-methods research have revealed that technology obstacles are seen as hindrances by pediatric nurses and physicians when it comes to utilizing telehealth for inter-facility transfers.^11,26^ Described telehealth technology barriers include challenges with navigating the telehealth platforms, large or stationary equipment restricting provider mobility, connectivity delays, and poor integration with professional interpreters.^27,28,11,26^ Providers described that their lack of confidence in using the telehealth equipment left them feeling vulnerable and “silly,” thus reducing their willingness to use telehealth.^11^ Additional workflow concerns expanded beyond the technology to include duplicative processes and unclear roles and expectations.^11,29^ Providers’ competing demands and limited availability to use telehealth exacerbate the importance of streamlined workflows that minimize provider burden.^29,30^

The insight that the intervention needs to equitably benefit diverse populations is another finding from the inspiration phase that is consistent with existing telehealth research. Telehealth has the potential to enhance equitable access to healthcare services by addressing barriers related to geography, time, and finances.^31^ However, despite its promise, this resource has exacerbated care access inequities for socially marginalized groups.^32^ Under-resourced populations have disproportionately lower access to telehealth services.^33–36^ Research focusing on the issues and dimensions that impact the reach and adoption of telehealth among diverse groups is critically needed.^37,38^ Research emphasizes that individuals with a language preference other than English experience reduced access to telehealth services in comparison to those with English language preference.^34^ Earlier studies also describe low digital literacy and limited Internet access as additional barriers restricting its accessibility and adoption.^39^ Designing telehealth interventions that equitably benefit diverse populations is a commonly shared priority, and the use of HCD approaches is a strategy that can be used to achieve this important goal.

This study focused on designing a nurse-to-family telehealth intervention to be used during pediatric transfers, which is an understudied application of telehealth. The body of literature on pediatric inter-facility telehealth research mostly examines physician telehealth use.^40–43^ The limited research studying telehealth use by nurses has examined nurse-to-nurse telehealth use for the inter-facility handoff.^44^ The potential benefits of our proposed nurse-to-family telehealth intervention are supported by existing research that suggests parents of transferring children seek information that nurses are well-positioned to provide, such as logistical information and what to expect at the post-transfer hospital.^15^ The findings from the inspiration phase of this present study are consistent with this prior research and further support the promise of this nurse-to-family telehealth intervention. Our parent participants shared that the intervention would help them understand the logistics surrounding the transfer process and procedures. Furthermore, our participants explained how the intervention could provide support to parents, mitigate parent stress, strengthen parent–provider relationships, and better prepare the nurses for the child's arrival. Future research testing the impact of nurse-to-family telehealth can include outcomes identified by our participants as potential benefits, including parent stress, parent–provider relationships, and nurse preparedness.

The next step from this present study is to conduct the nurse-to-family telehealth feasibility and pilot trial.^25^ Consistent with HCD principles, our future implementation phase will engage stakeholders throughout the trial. In doing so, likely benefits include improved protocol adherence, external validity, and ultimate uptake of the evidence into practice.^45,46^ Briefly, stakeholders will assist with trainings during the pre-trial preparation phase, site visits during the intervention period, troubleshooting of challenges that arise, data interpretation, and disseminating trial findings.

This study is not without limitations. While recruitment for the interviews and the stakeholder design workshop purposively sought to include a diverse range of roles and experiences, the participants were limited to those with English proficiency. We also limited participants to adults and thus did not obtain the perspectives of the pediatric patients. Another limitation was that the setting of this study was at a single telehealth program at a children's hospital. Although we included participants from only one post-transfer hospital, we included participants from three referring ED sites. Nevertheless, this study had a local focus; thus the findings from this study may not be generalizable to other hospitals. Additionally, this HCD process included the perspectives of 33 interview participants, 22 workshops participants, and feedback from 67 other stakeholders. The insights from these collective 122 individuals might not be representative of other individuals. Despite these limitations, this HCD study provided valuable information that could be considered by others in designing nurse-to-family telehealth interventions for pediatric inter-facility transfers.

## Conclusion

The use of HCD phases facilitated stakeholder engagement in developing a nurse-to-family telehealth intervention for pediatric inter-facility transfers. This telehealth intervention will be tested in an implementation phase as a feasibility and pilot trial. The insights and strategies identified in this study can be adapted to meet the local needs and contexts of other hospitals seeking to improve effective communication and engagement among providers and parents during inter-facility transfers.
